# Caregiver substance use among families in the U.S. child welfare system: national prevalence estimates

**DOI:** 10.3389/fpsyt.2025.1620676

**Published:** 2025-07-21

**Authors:** Susan Yoon, Charis Stanek, Juan Lorenzo Benavides, Taylor Napier, Yujeong Chang, Choong Rai Nho

**Affiliations:** ^1^ The Ohio State University, College of Social Work, Columbus, OH, United States; ^2^ Ewha Womans University, College of Social Sciences, Department of Social Welfare, Seoul, Republic of Korea

**Keywords:** parental substance use, alcohol use, drug use, child welfare system, child maltreatment, child abuse and neglect, prevalence, co-occurring family violence and substance use

## Abstract

**Introduction:**

Understanding the prevalence of caregiver substance use among families affected by the child welfare system can inform prevention and intervention efforts to reduce co-occurring caregiver substance use and child maltreatment. This study examined the national prevalence estimates of alcohol and drug dependence among caregivers affected by the U.S. child welfare system and explored variations based on child and caregiver characteristics.

**Methods:**

A secondary data analysis was conducted using the most recent data from the National Survey of Child and Adolescent Well-being (NSCAW-III).

**Results:**

Results indicated that approximately 8% of caregivers met criteria for alcohol dependence and 3% for drug dependence, with about 11% reporting either alcohol or drug dependence. For both alcohol dependence and drug dependence, higher prevalence rates were observed among White caregivers, male caregivers, caregivers of male children, and caregivers who were experiencing domestic violence and/or depression. Distinct patterns emerged in the prevalence rates of alcohol versus drug dependence across caregiver income, education, employment status, and the number of children’s out-of-home placements.

**Discussion:**

Our findings highlight nuanced differences between alcohol and drug dependence and point to the need for targeted and contextually responsive programs that address the complex intersection of caregiver substance use and child maltreatment.

## 1 Introduction

Caregiver substance use is a significant risk factor for child maltreatment, considering that it can impair caregivers’ ability to provide safe and stable care for their children. Substance use among parents has been linked to negative child welfare outcomes, including an increased risk of child abuse and neglect, higher rates of child removal, and lower rates of family reunification (1–3). Currently, national data on the prevalence of caregiver substance use among families involved with the child welfare system remain limited. Understanding the scope of this issue is critical for informing prevention and intervention efforts aimed at reducing parental substance use and co-occurring child maltreatment. Using the most recent data from the National Survey of Child and Adolescent Well-being (NSCAW-III), we examined the prevalence rate of caregiver substance use (alcohol dependence, drug dependence) among families affected by the child welfare system. Additionally, we explored differences in substance use prevalence based on child and caregiver characteristics.

### 1.1 Caregiver substance use and child maltreatment

Caregiver substance dependence, or caregivers’ reliance on alcohol or drugs to navigate daily life, presents an ongoing public health problem in the United States. More than 20 million people meet criteria for a substance use disorder (4) and research highlights parental substance use as one of the most common reasons why families are reported to the child welfare system (5, 6). Indeed, rates of child removal due to caregiver substance use have increased at an alarming rate in the past 20 years (7).

Past research documents associations between caregiver substance use and child maltreatment (3). Specifically, caregiver alcohol dependence is linked with more neglectful parenting (8), inconsistent discipline (9), and a heightened risk for physical abuse (10). Similarly, caregivers who endorsed drug use in the past year reported a higher frequency of physical and emotional abuse compared to caregivers who did not use drugs (11). Research also shows associations between caregiver drug use and higher rates of child neglect (11).

In addition, caregiver substance use predicts child welfare outcomes. Evidence suggests that children whose parents use substances are more likely to enter or re-enter foster care (1, 2), have a greater number of placements (12), and are less likely to reunify with their parents (13, 14). These findings are even stronger for children whose caregivers use both alcohol and other drugs (15). Moreover, children who experience maltreatment and live with a parent who uses substances often report poorer developmental outcomes (e.g., more depressive symptoms, aggression, and personal substance use) compared to children who experienced maltreatment but did not have a parent with substance use disorder (16, 17).

Challenges quantifying the national prevalence rates of substance use among parents reported to child welfare continue to plague the field (18). Past literature highlights inconsistencies in the reporting methods used to document caregiver substance use in child welfare reports, variable definitions of substance use in the literature (e.g., spectrum of use versus clinical criteria, differing types of substances explored), and a lack of standardized national data collection (18, 19). Findings across studies are also highly variable (19). Meinhofer and Angleró-Díaz (20) examined data from the Adoption and Foster Care Analysis and Reporting System, that gathers information on all children in foster care across the United States. Authors found that 23.38% of the entries between the years of 2000 and 2017 were attributable to parental substance use, representing a significant increase from 2000 (14.53%) to 2017 (36.26%). In this study, caregiver drug use was defined as, “the principal caretaker’s recurrent and lasting use of drug,” and drug use was listed as the reason for child removal. Other research using data from the National Surveys on Drug Use and Health from 2009 to 2014 suggests that 12.3% of children under the age of 17 lived with a caregiver with a substance use disorder (SUD; inclusive of alcohol and other drugs; 21). However, this study did not capture the percentage of children living with caregivers who may have dependence on alcohol or drugs but might not meet clinical criteria for a SUD. Using state-reported data, the U.S. Department of Health & Human Services, Children’s Bureau (22) reported that caregiver alcohol misuse was identified as a risk factor in 16.2% of substantiated child maltreatment cases, while caregiver drug misuse was noted in 24.9% of such cases. Yet, this data is also limited due to some states combining reports of alcohol and drug use into one variable, and others failing to systematically document substance use in child maltreatment reports (e.g., parental substance use is documented as “other”). Further, some studies attempting to ascertain accurate prevalence rates of caregiver substance use have done so by focusing on one specific type of substance. For example, Bullinger and Wing (23) found that the number of children living with parents with an opioid use disorder was 548,000 in 2017 compared to 432,000 in 2002, using data from the National Survey of Drug Use and Health. The same study also reported a 200% increase in children living with a parent who uses heroin across the same time period. While these studies provide some guidance related to the prevalence of caregiver substance use in cases reported to child welfare, more up-to-date information regarding the prevalence of both alcohol dependence and drug dependence among families affected by the child welfare system is critically needed.

### 1.2 Factors associated with caregiver substance use

Caregiver substance use in the child welfare system rarely occurs in isolation; instead, it is intertwined with a constellation of socioeconomic, psychological, and family challenges. For example, families affected by caregiver alcohol or drug use may also experience economic hardship, as chronic economic strain and unstable housing elevate parental stress and can precipitate or exacerbate substance use problems (24, 25). Caregivers with substance use disorder also frequently experience co-occurring mental health problems, such as depression, which compound the difficulty of providing consistent care to children (25, 26). Domestic violence is another risk factor that tends to co-occur with substance use in a vicious cycle of stress and conflict, further undermining family stability and safety (18, 27). Additionally, caregiver demographics shape these risk dynamics. Research indicates that younger parents, parents with lower levels of education, and unemployed parents are disproportionately represented among families affected by the child welfare system and substance use, reflecting broader socio-demographic vulnerabilities (26, 28). Taken together, prior research has suggested that families affected by the child welfare system who struggle with alcohol and drug dependence typically experience multiple overlapping adversities and the severity of caregiver substance use might be associated with other risk factors or family characteristics (25, 26).

### 1.3 The current study

Although prior research has established a significant association between caregiver substance use and child maltreatment (29), national data on the prevalence of parental alcohol and drug dependence among families affected by the child welfare system remain lacking. Illuminating the scope of this issue can inform policy and practice aimed at reducing the co-occurrence of caregiver substance use and child maltreatment and promoting the health and well-being of families affected by substance dependence. The primary aim of this study was to provide national estimates of alcohol and drug dependence among caregivers affected by the child welfare system. Specifically, we addressed two research questions: (1) What are the prevalence rates of alcohol and drug dependence among caregivers in the U.S. child welfare system? and (2) How do these rates vary based on child characteristics (e.g., age, sex) and caregiver characteristics (e.g., race/ethnicity, age, gender, education, depression, income, domestic violence)?

## 2 Method

### 2.1 Data source and sample

Data for the current study were derived from the third cohort of the National Survey of Child and Adolescent Well-Being (NSCAW-III), a nationally representative longitudinal study of children and families affected by the child welfare system (30). NSCAW-III builds upon two previous cohorts—NSCAW-I (data collected from 1999 to 2007) and NSCAW-II (data collected from 2008 to 2012). Data collection for NSCAW-III took place between July 2017 and September 2021, using a stratified, two-stage sampling approach (31). First, U.S. counties or clusters of neighboring counties were selected as primary sampling units, followed by the selection of individual children within these areas. The sample comprises approximately 3,300 children aged from birth to 17.5 years at the time of sampling. To ensure adequate representation, the study oversampled infants, children receiving child welfare services, and adolescents (ages 12-17) in out-of-home placements. Unlike earlier cohorts, NSCAW-III expanded its inclusion criteria beyond children involved in maltreatment investigations to also capture those who entered the child welfare system through alternative pathways, such as juvenile justice or human trafficking cases. Data collection primarily involved in-person interviews with children, caregivers, and caseworkers, primarily focusing on child safety, permanency, and well-being. Due to the COVID-19 pandemic, in-person interviews were suspended in March 2020, with caseworker interviews temporarily shifting to telephone administration in 2021 before in-person data collection resumed in June 2021. All NSCAW III Wave 1 permanent primary caregivers (i.e., birth parents, adoptive parents, or voluntary kin caregivers not receiving child welfare payments) were included in the present study. In the current study, the unweighted sample size was 2,701, and the total weighted sample size was 2,486,298.

### 2.2 Measures

#### 2.2.1 Alcohol and drug dependence

Alcohol dependence was measured using the Alcohol Use Disorders Identification Test (AUDIT), developed by the World Health Organization, which has shown good psychometric properties in general populations (32). The AUDIT has 10 items (e.g., “How often do you have a drink containing alcohol?”, “Have you or someone else been injured because of your drinking?”). The alcohol dependence cut-off was determined by caregivers who endorsed a 5 or > on the scale based on scholarly recommendation (33). Drug dependence was measured using the Drug Abuse Screening Test (DAST). Caregivers responded to the 20 items (e.g., “In the past 12 months, have you had blackouts or flashbacks due to drug use?”, “In the past 12 months, have you abused prescription drugs?”) on the DAST. The DAST has shown moderate to good reliability and validity in previous populations (34). The drug dependence cut-off was also determined by caregivers who endorsed 5 or > on the scale.

#### 2.2.2 Child characteristics

Child age was measured as a continuous variable in terms of years of age. Child sex at birth was measured as a binary variable (1 = boy, 2 = girl). The number of days in out-of-home care was measured as a continuous variable. A total number of out-of-home placements was also measured as a continuous variable.

#### 2.2.3 Caregiver characteristics

Caregiver age was measured as a continuous variable (i.e., years of age). Gender was measured as a binary variable (1 = male, 2 = female). Race/ethnicity was a categorical variable (1 = Black, 2 = White, 3 = Hispanic, 4 = Other). Education was measured as an ordinal variable (1 = less than high school, 2 = high school, 3 = more than high school). Employment was measured as a categorical variable (1 = full-time, 2 = part-time, 3 = unemployed, 4 = do not work, 5 = other). Employment was recoded as a binary variable (0 = unemployed, 1 = employed) for the purpose of analysis.

Family income was measured by the percent of the federal poverty level (1 = less than 50%, 2 = 50% to less than 100%, 3 = 100% through 200%, and 4 = greater than 200%). Family income was recoded as a binary variable (0 = below 100% of the federal poverty level, 1 = 100% or greater % of the federal poverty level). Caseworker reports of caregiver depression and domestic violence were assessed using a risk assessment completed by caseworkers. Caregiver depression was measured as a binary variable (0 = no depression within the past month, 1 = depression within the past month). Domestic violence was measured as a binary variable (0 = no domestic violence within the past month, 1 = domestic violence within the past month).

#### 2.2.3 Data analysis

Descriptive statistics were used to characterize the sample and to calculate prevalence rates of alcohol and drug dependence. Chi-squares were used to examine bivariate associations between categorical variables and caregiver alcohol and drug dependence. Independent samples *t*-tests were used to examine bivariate associations between continuous variables and caregiver alcohol and drug dependence. Statistically significant associations were determined at the *p* <.001 level, considering the study’s large sample size. Cramer’s V values were used to interpret effect sizes from Chi-square analyses. Cohen’s *d* values were used to interpret effect sizes from *t*-tests. Analyses were conducted in SPSS version 28.0 (35). A national sample weight was used to calculate all analyses.

## 3 Results

### 3.1 Prevalence rates of alcohol and drug dependence

**Table 1** displays national estimates of prevalence rates of alcohol and drug dependence among primary caregivers in the U.S. child welfare system. The number of caregivers who met the criteria for alcohol dependence was 174,390, representing about 8% of the caregivers in the child welfare system. The number of caregivers who met the criteria for drug dependence was 71,620, representing 3% of the caregivers in the child welfare system. Approximately 11% met either criterion, and 1% met the criteria for both.

**Table 1:** Prevalence rates of alcohol dependence and drug dependence.

	n	%
Alcohol dependence	174,390	8%
Drug dependence	71,620	3%
Either alcohol dependence or drug dependence	234,679	11%
Both alcohol dependence and drug dependence	11,331	1%

### 3.2 Prevalence of alcohol dependence across child and caregiver characteristics

There was a statistically significant association between caregiver race and meeting the criteria for alcohol dependence, χ^2^ (3) = 2138.603, *p* <.001 (**Table 2**). Caregivers who met the criteria for alcohol dependence were disproportionately White (8.2%) compared to Black (6.3%), Hispanic (7.1%), or “Other” (7.0%). There was also a significant association between alcohol dependence and caregiver education, χ^2^ (2) = 824.048, *p* <.001. The largest percentage of caregivers who met the criteria for alcohol dependence had completed more than a high school education (8.3%) compared to those with a high school diploma (7.2%) or less than high school education (7.3%). There was a statistically significant association between meeting the criteria for alcohol dependence and federal poverty level reported, χ^2^ (1) = 8618.280, *p* <.001. Caregivers who met the criteria for alcohol dependence were more likely to have income at or above the federal poverty level (9.5%), compared to those who did not (6.2%). The relationship between alcohol dependence and employment status was also significant, χ^2^ (1) = 21843.565, *p* <.001. Caregivers who met the criteria for alcohol dependence were more likely to be employed (9.7%) compared to those who were not (4.5%). Additionally, caregivers who met the criteria for alcohol dependence were significantly more likely to be married, χ^2^ (1) = 6554.060, *p* <.001 (8.5% vs. 5.4%), to have experienced major depression within the last month, χ^2^ (1) = 8339.459, *p* <.001 (17.0% vs. 7.3%) and more likely to have experienced domestic violence in the last month, χ^2^ (1) = 107.191, *p* <.001 (9.0% vs. 8.2%). Finally, children whose primary caregiver met the criteria for alcohol dependence were more likely to have been in fewer out-of-home placements than those whose caregiver did not meet the criteria for alcohol dependence.

**Table 2:** Chi-Square results for family characteristics and alcohol dependence.

Variable	Alcohol dependence		*X* ^2^	*df*	*p*	Cramer’s *V*
	No	Yes				
Caregiver race						
*Black*	476,204 (93.7%)	32,143 (6.3%)	2138.603	3	<.001	.03
*Hispanic*	437,654 (92.9%)	33,206 (7.1%)				
*White*	1,119,716 (91.8%)	100,519 (8.2%)				
*“Other”*	94,990 (93.0%)	7156 (7.0%)				
Caregiver sex						
*Man*	1,942,214 (87.6%)	27,618 (12.4%)	8465.492	1	<.001	.06
*Woman*	1,942,901 (93.0%)	146,722 (7.0%)				
Child sex						
*Boy*	1,067,632 (91.5%)	98,958 (8.5%)	2972.361	1	<.001	.04
*Girl*	1,069,482 (93.4%)	75,432 (6.6%)				
Caregiver education						
*< High school*	392,378 (92.7%)	30854 (7.3%)	824.048	2	<.001	.02
*High school*	997,714 (92.8%)	77,121 (7.2%)				
*> High school*	738,259 (91.7%)	66,415 (8.3%)				
Income						
*< Federal poverty level*	1,142,130 (93.8%)	74,855 (6.2%)	8618.280	1	<.001	.06
≥*Federal poverty level*	908,734 (90.5%)	95,187 (9.5%)				
Employment						
*Employed*	1,207,817 (90.3%)	130,251 (9.7%)	21843.565	1	<.001	.10
*Unemployed*	929,298 (95.5%)	44,139 (4.5%)				
Marital Status						
*Single parent*	646,366 (94.6%)	36,811 (5.4%)	6554.060	1	<.001	.05
*Married*	1,485,831 (91.5%)	137,579 (8.5%)				
Active depression						
*Yes*	53,010 (83.0%)	10,830 (17.0%)	8339.459	1	<.001	.06
*No*	2,082,725 (92.7%)	163,560 (7.3%)				
Active domestic violence						
*Yes*	153,850 (91.0%)	15,153 (9.0%)	107.191	1	<.001	.01
*No*	1,180,221 (91.8%)	105,795 (8.2%)				

Cramer’s V =.10 is a small effect, .30 is a moderate effect, and .50 is a large effect.

### 3.3 Prevalence of drug dependence across child and caregiver characteristics

There was a statistically significant association between caregiver race and drug dependence, χ^2^ (3) = 32910.770, *p* <.001 (**Table 3**). There were disproportionately more White caregivers (5.3%) who met the criteria for drug dependence compared to 2.0% who were Hispanic, 1% in the “Other” racial category, and <1% who were Black. There was also a statistically significant association between caregiver education and drug dependence, χ^2^ (2) = 3925.706, *p* <.001. There was a disproportionality higher number of caregivers who did not complete a high school education (4.8%) compared to those who completed high school (3.1%) or completed more than high school education (2.7%). There was a statistically significant association between meeting the criteria for drug dependence and federal poverty level reported, χ^2^ (1) = 523.441, *p* <.001. Caregivers who met the criteria for drug dependence were more likely to live under the federal poverty line (3.5%). The relationship between drug dependence and employment status was also significant, χ^2^ (1) = 12855.813, *p* <.001. Caregivers who met the criteria for drug dependence were less likely to be employed. Additionally, caregivers who met the criteria for drug dependence were significantly less likely to be married, χ^2^ (1) = 7718.491, *p* <.001 (2.6% vs 4.9%), more likely to have experienced major depression within the last month, χ^2^ (1) = 3198.017, *p* <.001 (7.2% vs. 3.1%) and more likely to have experienced domestic violence in the last month, χ^2^ (1) = 3836.517, *p* <.001 (6.6% vs. 3.5%). Effect sizes across Chi-square tests were negligible or small for all models (see **Tables 2**, **3**). Finally, caregivers who had a drug dependence were, on average, younger than caregivers who did not. The Cohen’s d was .57, indicating a moderate effect size. Children whose caregiver experienced drug dependence were more likely to be younger than children whose primary caregiver did not have a drug dependence (**Table 4**).

**Table 3:** Chi-Square results for family characteristics and drug dependence.

Variable	Drug dependence		*X* ^2^	*df*	*p*	Cramer’s *V*
	No	Yes				
Caregiver race						
*Black*	484,441 (99.7%)	1234 (.3%)	32910.770	3	<.001	.12
*Hispanic*	439,045 (98.0%)	8964 (2.0%)				
*White*	1,095,594 (94.7%)	60,810 (5.3%)				
*“Other”*	98,852 (99.4%)	612 (.6%)				
Caregiver sex						
*Male*	210,782 (98.3%)	3657 (1.7%)	1815.076	1	<.001	.03
*Female*	1,916,712 (96.6%)	67,963 (3.4%)				
Child sex						
*Male*	1,067,155 (96.9%)	34,308 (3.1%)	141.228	1	<.001	.01
*Female*	1,060,339 (96.6%)	37,312 (3.4%)				
Caregiver education						
*< High school*	376,648 (95.2%)	19,155 (4.8%)	3925.706	2	<.001	.04
*High school*	990,943 (96.9%)	31,320 (3.1%)				
*> High school*	751,176 (97.3%)	21,108 (2.7%)				
Income						
*< Federal poverty level*	1,116.164 (96.5%)	40,770 (3.5%)	523.441	1	<.001	.02
*≥ Federal poverty level*	941,562 (97.0%)	28,760 (3.0%)				
Employment						
*Employed*	1,254,357 (97.9%)	27,014 (2.1%)	12855.813	1	<.001	.08
*Unemployed*	873,136 (95.1%)	44,606 (4.9%)				
Marital Status						
*Single parent*	612,367 (95.1%)	31,527 (4.9%)	7718.491	1	<.001	.06
*Married*	1,510,755 (97.4%)	40,053 (2.6%)				
Active depression						
*Yes*	58,959 (92.8%)	4564 (7.2%)	3198.017	1	<.001	.04
*No*	2,067,155 (96.9%)	67.057 (3.1%)				
Active domestic violence						
*Yes*	153,850 (93.4%)	10,820 (6.6%)	3836.517	1	<.001	.05
*No*	1,177,011 (96.5%)	42,087 (3.5%)				

Cramer’s V =.10 is a small effect, .30 is a moderate effect, and .50 is a large effect.

**Table 4:** T-test results for child and caregiver characteristics and substance use dependence.

Variable	No alcohol dependence	Alcohol dependence	*t*	*df*	Cohen’s d
	Mean (SD)	Mean (SD)			
Caregiver Age	35.80 (10.12)	34.97 (8.68)	37.752*	214104	.08
Child Age	8.19 (4.86)	8.34 (5.14)	-12.119*	200678	-.03
Total Days Out of Home	36.85 (177.81)	11.34 (60.40)	110.675*	354494	.15
Total OOH Placements	.13 (.36)	.04 (.19)	144.540*	205997	.26

	No drug dependence	Drug dependence	t	*df*	Cohen’s d
	Mean (SD)	Mean (SD)			
Caregiver Age	35.90 (9.94)	30.27 (6.15)	234.958*	84752	.57
Child Age	8.23 (4.85)	6.28 (4.55)	112.543*	77204	.40
Total Days Out of Home	34.11 (176.44)	46.62 (109.15)	-25.385*	65665	-.07
Total OOH Placements	.12 (.35)	.17 (.39)	-32.291*	57432	-.16

*p<.001; OOH, out-of-home; Cohen’d is used to determine effect sizes. Based on Cohen (36), d |.20| indicates a small effect, |.50| indicates a moderate effect, and >|.80| indicates a large effect.

## 4 Discussion

The main purpose of this study was to provide national estimates of alcohol and drug dependence among caregivers affected by the child welfare system. We found that approximately 8% of caregivers met criteria for alcohol dependence and 3% for drug dependence among families affected by the U.S. child welfare system. About 11% of caregivers reported either alcohol or drug dependence. The higher prevalence of alcohol dependence compared to drug dependence should be interpreted with caution, as it may reflect lower stigma and greater social acceptability associated with alcohol use, rather than true differences in prevalence. It is possible that caregivers were more forthcoming about alcohol use than drug use due to the lower stigma regarding alcohol use (37).

The prevalence rates of alcohol (8%) and drug (3%) dependence found in the current study are somewhat lower than those reported in previous studies, which have varied widely depending on data sources and measurement approaches (19). For example, using general population data, one study found that 12.3% of children in the U.S. lived with a caregiver with SUD (21). Higher prevalence rates have been reported in child welfare samples, including 23.4% of foster care entries attributed to parental substance use (20). Further, a study that used data reported by state child welfare agencies found that alcohol misuse and drug misuse were identified as risk factors in 16.2% and 24.9% of substantiated child maltreatment cases, respectively (22). The relatively lower rates observed in our study may reflect differences in sampling strategies, operational definitions (e.g., clinical dependence versus broader substance use), or the reliance on caregiver self-report, which may be affected by underreporting due to stigma or fear of legal consequences, such as child removal or loss of custody. These discrepancies highlight the need for consistent and standardized methods of collecting national data on caregiver substance use to better inform child welfare policy and practice.

The analysis of prevalence rates of alcohol and drug dependence across child and caregiver characteristics revealed some interesting patterns, highlighting both overlapping and distinct correlates associated with each type of substance. For alcohol dependence, higher prevalence rates were found among White caregivers, male caregivers, caregivers of male children, caregivers with more than a high school education, those with income at or above the federal poverty level, employed caregivers, those with depression, those experiencing domestic violence, and those whose children have had fewer out-of-home placements. For drug dependence, higher prevalence rates were found among White caregivers, younger caregivers, female caregivers, caregivers with female children or younger children, caregivers with less than a high school education, caregivers with income below the federal poverty level, unemployed caregivers, those with depression, and those experiencing domestic violence.

For alcohol dependence, higher prevalence rates were observed among White caregivers, male caregivers, and caregivers of male children. These results are in line with findings from previous studies that found higher rates of substance use among men compared to women, and among White individuals compared to Black individuals (38, 39). However, more recent studies reveal the complexities of these connections (40). For instance, despite lower overall prevalence rates, women may experience a more rapid progression from substance use to dependence (a phenomenon known as “telescoping effect”; 41). Women also often report greater substance-related impairment (42) and face more barriers to accessing treatment services (43). Building upon the results of the current study, future research should unpack the nuanced and complex relationships between demographic characteristics and substance dependence among families affected by the child welfare system.

Prevalence rates of alcohol and drug dependence were also higher among caregivers who were experiencing domestic violence and/or depression. Previous studies have reported high rates of co-occurrence among depression, domestic violence, and substance use (44). Caregivers who experience depression and/or domestic violence may use alcohol or drugs to self-medicate their physical and emotional pain and to cope with trauma and psychological distress (45). While no causal inference can be made in this study regarding the associations among domestic violence, depression, and alcohol and drug dependence, our results suggest the need for comprehensive risk assessments and better integration and collaboration across domestic violence, mental health, and substance use treatment systems.

Notably, distinct patterns emerged in the prevalence rates of alcohol versus drug dependence across caregiver income, education, employment status, and the number of children’s out-of-home placements. Specifically, alcohol dependence was more prevalent among caregivers with higher incomes, higher educational attainment (beyond high school), those who were married, and those who were employed. Conversely, drug dependence was more prevalent among caregivers with lower incomes (i.e., below the federal poverty level), lower education levels (high school or less), those who were single parents, and those who were unemployed. There are several possible explanations for these seemingly paradoxical findings. First, alcohol is often viewed as a socially acceptable substance, especially in the context of social drinking, whereas there is greater stigma associated with drug use (37). Employment may also increase opportunities for alcohol use through workplace-related social events that involve social drinking. Additionally, being married and employed may be associated with higher income, which could increase access to alcohol, particularly in settings where drinking can be costly (e.g., bars and restaurants). Consistent with our findings, prior research has found a positive association between SES and alcohol dependence (46).

Another unexpected finding, though small in effect size, was the higher prevalence of alcohol dependence among caregivers whose children had fewer out-of-home placements. This seemingly counterintuitive result may, as discussed above, reflect the lower stigma associated with alcohol use, which could shape how child welfare systems respond. For example, caseworkers may view alcohol use as more socially acceptable and, therefore, less indicative of a caregiver’s inability to provide adequate care for their child, especially when other factors, such as stable income and employment, suggest that the caregiver can financially support the child. While placement decisions are complex and determined by multiple factors, prior research has shown that perceptions of available family resources can influence these decisions (47).

### 4.1 Limitations

The study has several limitations. First, we used cross-sectional data, which limited our ability to infer any causal relationships between prevalence rates of substance dependence and various child, caregiver, and family factors. Additionally, we could not examine the changes in the prevalence of caregiver alcohol and drug dependence over time. Future research should employ longitudinal designs to capture developmental trajectories of substance dependence. Second, substance use was assessed through caregiver self-report, which may be subject to social desirability bias. Incorporating objective measures, such as biological testing or clinical records, could enhance the validity and reliability of these assessments. Third, the quantitative nature of the data limited our ability to explore the underlying reasons for caregiver substance dependence within the child welfare system. Qualitative research is needed to provide a more nuanced understanding of these complex dynamics. Despite these limitations, this study makes an important contribution by offering updated national estimates of alcohol and drug dependence among caregivers involved with the child welfare system.

### 4.2 Implications

Our findings have important implications for future child welfare research, policy, and practice. From a research perspective, the variability in reported prevalence rates of caregiver substance use across study findings, including our findings from the current study, underscores the need for more consistent, standardized, and nationally representative methods to assess and document caregiver substance use in child welfare populations. In terms of policy, these findings point to the need for increased funding and support for programs and research that address the intersection of caregiver substance use and child maltreatment. Notably, even using a conservative measure with strict cutoffs for alcohol and drug dependence, we found that approximately 11% of caregivers met criteria for either alcohol or drug dependence. This finding highlights the pervasive and serious nature of this issue. For practitioners, the findings suggest the need for evidence-based interventions that support families affected by co-occurring parental substance use and child welfare involvement. One such model that may offer a promising framework for service delivery is Sobriety, Treatment, and Recovery Teams (START; 48). The START model is a children-services-led initiative designed to provide rapid access to substance use treatment, intensive case management, and family peer mentor services to address these dual challenges. START has been implemented in multiple states across the United States and has shown promising outcomes in enhancing child and family well-being (48, 49). Additionally, our findings highlight nuanced differences between alcohol and drug dependence, pointing to the importance of implementing targeted, contextually responsive interventions when working with families facing these complex challenges.

## Data Availability

The original contributions presented in the study are included in the article/supplementary material, further inquiries can be directed to the corresponding author/s.

## References

[1] Maluccio AN Ainsworth F. Drug use by parents: A challenge for family reunification practice. Children Youth Serv Rev. (2003) 25:511–33. doi: 10.1016/S0190-7409(03)00042-2

[2] Miller KA Fisher PA Fetrow B Jordan K. Trouble on the journey home: Reunification failures in foster care. Children Youth Serv Rev. (2006) 28:260–74. doi: 10.1016/j.childyouth.2005.03.010

[3] Neger EN Prinz RJ. Interventions to address parenting and parental substance abuse: Conceptual and methodological considerations. Clin Psychol Rev. (2015) 39:71–82. doi: 10.1016/j.cpr.2015.04.004, PMID: 25939033 PMC4464898

[4] Children’s Bureau. The AFCARS report No. 26: Preliminary FY 2018 estimates as of August 22, 2019 (2019). Available online at: https://www.acf.hhs.gov/sites/default/files/cb/afcarsreport26.pdf.

[5] Children’s Bureau. Child maltreatment 2010. Washington, D.C: Administration on Children, Youth, and Families (2011). Available online at: https://www.acf.hhs.gov/archive/cb/resource/child-maltreatment-2010 (Accessed April 29, 2025).

[6] Children’s Bureau. Child maltreatment 2018 (2019). Available online at: https://www.acf.hhs.gov/sites/default/files/cb/cm2018.pdf (Accessed April 29, 2025).

[7] Sieger MHL. Reunification for young children of color with substance removals: An intersectional analysis of longitudinal national data. Child Abuse Negl. (2020) 108:104664. doi: 10.1016/j.chiabu.2020.104664, PMID: 32799013

[8] Connell-Carrick K. A critical review of the empirical literature: Identifying correlates of child neglect. Child Adolesc Soc Work J. (2003) 20:389–425. doi: 10.1023/A:1026099913845

[9] Taber-Thomas SM Knutson JF. Association between mothers’ alcohol use histories and deficient parenting in an economically disadvantaged sample. Child Maltreatment. (2021) 26:40–9. doi: 10.1177/1077559520925550, PMID: 32431161

[10] Freisthler B Gruenewald PJ. Where the individual meets the ecological: A study of parent drinking patterns, alcohol outlets, and child physical abuse. Alcoholism: Clin Exp Res. (2013) 37:993–1000. doi: 10.1111/acer.12059, PMID: 23316780 PMC3628976

[11] Kepple NJ. Does parental substance use always engender risk for children? Comparing incidence rate ratios of abusive and neglectful behaviors across substance use behavior patterns. Child Abuse Negl. (2018) 76:44–55. doi: 10.1016/j.chiabu.2017.09.015, PMID: 29032186

[12] Tracy EM Farkas KJ. Preparing practitioners for child welfare practice with substance-abusing families. Child Welfare. (1994) 73:57–68., PMID: 8299409

[13] Meyer AS McWey LM McKendrick W Henderson TL. Substance using parents, foster care, and termination of parental rights: The importance of risk factors for legal outcomes. Children Youth Serv Rev. (2010) 32:639–49. doi: 10.1016/j.childyouth.2009.12.011

[14] Akin BA Brook J Lloyd MH McDonald TP. Effect of a parenting intervention on foster care reentry after reunification among substance-affected families: A quasi-experimental study. Child Maltreatment. (2017) 22:194–204. doi: 10.1177/1077559517702743, PMID: 28393533

[15] Brook J McDonald T. The impact of parental substance abuse on the stability of family reunifications from foster care. Children Youth Serv Rev. (2009) 31:193–8. doi: 10.1016/j.childyouth.2008.07.010

[16] Smith VC Wilson CR. Families affected by parental substance use. Pediatrics. (2016) 138:e1–e13. doi: 10.1542/peds.2016-1575, PMID: 27432847

[17] Stein JA Leslie MB Nyamathi A. Relative contributions of parent substance use and childhood maltreatment to chronic homelessness, depression, and substance abuse problems among homeless women: Mediating roles of self-esteem and abuse in adulthood. Child Abuse Negl. (2002) 26:1011–27. doi: 10.1016/S0145-2134(02)00382-4, PMID: 12398858

[18] Young NK Boles SM Otero C. Parental substance use disorders and child maltreatment: Overlap, gaps, and opportunities. Child Maltreatment. (2007) 12:137–49. doi: 10.1177/1077559507300322, PMID: 17446567

[19] Seay K. How many families in child welfare services are affected by parental substance use disorders? A common question that remains unanswered. Child Welfare. (2015) 94:19., PMID: 26827475 PMC4894838

[20] Meinhofer A Angleró-Díaz Y. Trends in foster care entry among children removed from their homes because of parental drug us to 2017. JAMA Pediatr. (2019) 173:881–3. doi: 10.1001/jamapediatrics.2019.1738, PMID: 31305925 PMC6632152

[21] Lipari RN Van Horn SL. Children living with parents who have a substance use disorder. In: The CBHSQ report. Rockville, MD: Substance Abuse and Mental Health Services Administration (2017) ., PMID: 29144715

[22] U.S. Department of Health & Human Services Administration for Children and Families, & Children’s Bureau. Child maltreatment (2025). Available online at: https://www.acf.hhs.gov/cb/data-research/child-maltreatment (Accessed April 29, 2025).

[23] Bullinger LR Wing C. How many children live with adults with opioid use disorder? Children Youth Serv Rev. (2019) 104:104381. doi: 10.1016/j.childyouth.2019.06.016

[24] Wahler EA Otis MD. Social stress, economic hardship, and psychological distress as predictors of sustained abstinence from substance use after treatment. Subst Use Misuse. (2014) 49:1820–32. doi: 10.3109/10826084.2014.935789, PMID: 25050587

[25] Testa MF Smith B. Prevention and drug treatment. Future Children. (2009) 19:147–68. doi: 10.1353/foc.0.0033, PMID: 19719026

[26] Oliveros A Kaufman J. Addressing substance abuse treatment needs of parents involved with the child welfare system. Child Welfare. (2011) 90:25., PMID: 21950173 PMC4158612

[27] Choi S Ryan JP. Co-occurring problems for substance abusing mothers in child welfare: Matching services to improve family reunification. Children Youth Serv Rev. (2007) 29:1395–410. doi: 10.1016/j.childyouth.2007.05.013

[28] Dolan M Casanueva C Smith K Lloyd S Ringeisen H. NSCAW II Wave 2 report: Caregiver health and services. Washington, DC: US Department of Health and Human Services (2012).

[29] Ghertner R Waters A Radel L Crouse G. The role of substance use in child welfare caseloads. Children Youth Serv Rev. (2018) 90:83–93. doi: 10.1016/j.childyouth.2018.05.015

[30] Armstrong JM Dolan M Biemer P Ringeisen H Testa M Keeney J. *NSCAW III: Design overview, methodological challenges, and lessons learned from the baseline wave* (OPRE Report 2024-040). Washington, DC: Office of Planning, Research, and Evaluation, Administration for Children and Families, U.S. Department of Health and Human Services (2024).

[31] Casanueva C Armstrong JM Kluckman M Larrabee HM Ringeisen H. NSCAW III baseline report 2017-2022): *introduction to NSCAW III* (OPRE report 2024-024. Washington, DC: Office of Planning, Research, and Evaluation, Administration for Children and Families, U.S. Department of Health and Human Services (2024).

[32] Reinert DF Allen JP. The alcohol use disorders identification test (AUDIT): A review of recent research. Alcoholism. Clin Exp Res. (2002) 26:272–9. doi: 10.1111/j.1530-0277.2002.tb02534.x, PMID: 11964568

[33] Rumpf H-J Hapke U Meyer C John U. Screening for alcohol use disorders and at-risk drinking in the general population: Psychometric performance of three questionnaires. Alcohol Alcoholism. (2002) 37:261–8. doi: 10.1093/alcalc/37.3.261, PMID: 12003915

[34] Yudko E Lozhkina O Fouts A. A comprehensive review of the psychometric properties of the Drug Abuse Screening Test. J Subst Abuse Treat. (2007) 32:189–98. doi: 10.1016/j.jsat.2006.08.002, PMID: 17306727

[35] IBM Corp, N. *IBM SPSS statistics for windows.* In Version 25.0. NY: IBM corp Armonk (2017).

[36] Cohen J. The effect size. Statistical power analysis for the behavioral sciences. Hillsdale, NJ: Routledge (1988) p. 77–83.

[37] Johnson TP. Sources of error in substance use prevalence surveys. Int Scholarly Res Notices. (2014) 2014:923290. doi: 10.1155/2014/923290, PMID: 27437511 PMC4897110

[38] Compton WM Cottler LB Ben Abdallah A Phelps DL Spitznagel EL Horton JC. Substance dependence and other psychiatric disorders among drug dependent subjects: race and gender correlates. Am J Addict. (2000) 9:113–25. doi: 10.1080/10550490050173181, PMID: 10934573

[39] Vasilenko SA Evans-Polce RJ Lanza ST. Age trends in rates of substance use disorders across ages 18–90: Differences by gender and race/ethnicity. Drug Alcohol Depend. (2017) 180:260–4. doi: 10.1016/j.drugalcdep.2017.08.027, PMID: 28938183 PMC5757874

[40] McKee SA McRae-Clark AL. Consideration of sex and gender differences in addiction medication response. Biol sex Dif. (2022) 13:34. doi: 10.1186/s13293-022-00441-3, PMID: 35761351 PMC9235243

[41] Towers EB Williams IL Qillawala EI Rissman EF Lynch WJ. Sex/gender differences in the time-course for the development of substance use disorder: a focus on the telescoping effect. Pharmacol Rev. (2023) 75:217–49. doi: 10.1124/pharmrev.121.000361, PMID: 36781217 PMC9969523

[42] McHugh RK Votaw VR Sugarman DE Greenfield SF. Sex and gender differences in substance use disorders. Clin Psychol Rev. (2018) 66:12–23. doi: 10.1016/j.cpr.2017.10.012, PMID: 29174306 PMC5945349

[43] Tuchman E. Women and addiction: the importance of gender issues in substance abuse research. J Addictive Dis. (2010) 29:127–38. doi: 10.1080/10550881003684582, PMID: 20407972

[44] Mason R O’rinn SE. Co-occurring intimate partner violence, mental health, and substance use problems: a scoping review. Global Health Action. (2014) 7:24815. doi: 10.3402/gha.v7.24815, PMID: 25416321 PMC4240863

[45] Khantzian EJ. The self-medication hypothesis of substance use disorders: A reconsideration and recent applications. Harvard Rev Psychiatry. (1997) 4:231–44. doi: 10.3109/10673229709030550, PMID: 9385000

[46] KM Hasin DS. Socio‐economic status and problem alcohol use: the positive relationship between income and the DSM‐IV alcohol abuse diagnosis. Addiction. (2008) 103(7):1120–1130., PMID: 18494841 10.1111/j.1360-0443.2008.02218.x PMC3859240

[47] Sieracki JH Fuller AK Leon SC Bai GJ Bryant F. The role of race, socioeconomic status, and System of Care services in placement decision-making. Children Youth Serv Rev. (2015) 50:3–11. doi: 10.1016/j.childyouth.2014.12.013

[48] Huebner RA Willauer T Posze L. The impact of Sobriety Treatment and Recovery Teams (START) on family outcomes. Families Soc. (2012) 93:196–203. doi: 10.1606/1044-3894.4223

[49] Yoon S Ploss A Hutzel M Webb R Hatfield A Lee JY. Parenting attitudes and behaviors among parents involved with the child welfare system and affected by substance use disorders. Child Abuse Negl. (2024) 149:106657. doi: 10.1016/j.chiabu.2024.106657, PMID: 38262180

